# Polymeric Gel Systems Cytotoxicity and Drug Release as Key Features for their Effective Application in Various Fields of Addressed Pharmaceuticals Delivery

**DOI:** 10.3390/pharmaceutics15030830

**Published:** 2023-03-03

**Authors:** Veronika Smagina, Pavel Yudaev, Andrey Kuskov, Evgeniy Chistyakov

**Affiliations:** Mendeleev University of Chemical Technology of Russia, 125047 Moscow, Russia

**Keywords:** hydrogels, nanogels, drug delivery system, pharmaceutics, medications, eye diseases, dentistry, cancer

## Abstract

Modified polymeric gels, including nanogels, which play not only the role of a bioinert matrix, but also perform regulatory, catalytic, and transport functions due to the active fragments introduced into them, can significantly advance the solution to the problem of targeted drug delivery in an organism. This will significantly reduce the toxicity of used pharmaceuticals and expand the range of their therapeutic, diagnostic, and medical application. This review presents a comparative description of gels based on synthetic and natural polymers intended for pharmaceutical-targeted drug delivery in the field of therapy of inflammatory and infectious diseases, dentistry, ophthalmology, oncology, dermatology, rheumatology, neurology, and the treatment of intestinal diseases. An analysis was made of most actual sources published for 2021–2022. The review is focused on the comparative characteristics of polymer gels in terms of their toxicity to cells and the release rate of drugs from nano-sized hydrogel systems, which are crucial initial features for their further possible application in mentioned areas of biomedicine. Different proposed mechanisms of drug release from gels depending on their structure, composition, and application are summarized and presented. The review may be useful for medical professionals, and pharmacologists dealing with the development of novel drug delivery vehicles.

## 1. Introduction

Hydrogels are three-dimensional cross-linked polymer networks capable of adsorbing large volumes of water (the degree of swelling reaches several thousand percent) and imitating human tissues [1,2,3,4,5]. Hydrogels 20–30 nm in size (“nanogels”) are capable of incorporating hydrophobic drugs into the core and delivering them to their destination, providing their prolonged release. In particular, hydrogel delivery systems for enzymes [6], proteins [7], antibiotics [8,9,10,11], adenoviruses [12], anticancer drugs, for example, doxorubicin [13,14,15], paclitaxel [16], 5-fluorouracil [17,18,19], kaempferol [20], vincristine [21], oxaliplatin [22], cyclophosphamide [23], carmustine and curcumin [24], drugs for the treatment of skin diseases [25,26,27], anesthetics for the treatment of acute postoperative pain [28], antimicrobial silver nanoparticles and quantum dots for wound healing [29,30] are known and studied currently. The rate of release of these mentioned drugs from hydrogels is affected by the degree of cross-linking and the degree of swelling of the hydrogels.

Polymer hydrogels are able to respond to changes in the pH of the physiological environment and temperature, which makes it possible to create “smart” systems for controlled drug delivery [31,32,33]. For application in pharmacology and biomedicine, polymer hydrogels must have biocompatibility and mechanical strength [34], low toxicity, and good swelling behavior in physiological media [35]. Therefore, the range of polymers used for the manufacture of hydrogels is limited. These include natural and synthetic polymers, in particular, poly-N-vinylpyrrolidone [36], polyoxazolines and copolymers of oxazolines [37,38,39], polysaccharides [40], polyurethanes [41], poly-N-isopropylacrylamide [42], polyacrylic acid [43] and others.

Hydrogels are obtained by polymers cross-linking using physical and chemical methods. Chemical cross-linking method often involves the use of toxic cross-linking agents and catalysts which is the limiting factor for biomedical and pharmaceutical application of the prepared systems. For example, toxic glutaraldehyde and hydrochloric acid are used to crosslink polyvinyl alcohol [44]. The most promising method of crosslinking is radiation crosslinking of polymers using radioactive isotopes of cobalt ^60^Co, since there is no need to remove toxic crosslinkers in the process of radiation crosslinking [45,46,47,48,49,50]. Radiation crosslinking makes it possible to obtain soft and transparent gels at room temperature within a short period of time. At the same time, exposure to radiation ensures sterilization of the gel without the need for autoclaving at high temperatures, since some components of hydrogels are sensitive to high temperatures.

According to the PubMed database, more than 200 review articles have been published over the past two years on polymer hydrogels for biomedical applications, for example, hydrogels for the manufacture of wound dressings with drug loading and wound healing effect [51], nanogels for the treatment of brain cancer [52], hydrogels for the treatment of periodontal diseases [53], mucoadhesive gel systems for the delivery of buccal preparations [54], in situ nasal gels for the treatment of neurological disorders [55], self-assembled polysaccharide-based nanogels [56], hydrogel stability studies [57]. This particular review focuses on the comparative characterization of polymer hydrogels in terms of their toxicity to cells and the drug release rate from nano-sized gel systems which are the most important features for gels pharmaceutical and biomedical applications. This paper provides an analysis of works on gel drug delivery systems in various fields of biomedicine which were published in 2021–2022.

## 2. Gels for the Treatment of Diseases of the Intestines and Skin

**Figure 1:**
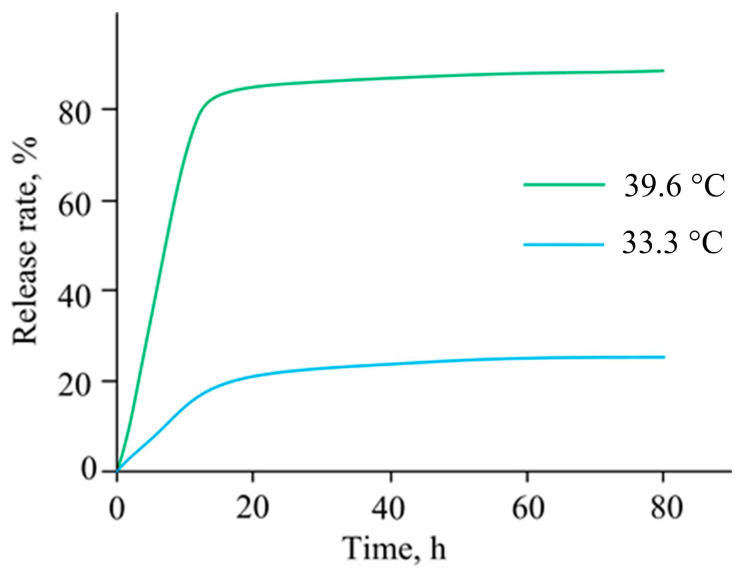
Release of tetracycline from dual-network hydrogels at different temperatures.

The tetracycline antibiotic is used in therapy to treat bacterial intestinal infections. The incorporation of tetracycline into the structure of a dual-network hydrogel based on carboxymethyl cellulose and a block copolymer of N-isopropylacrylamide and acrylamide provides a controlled prolonged release of tetracycline in the small intestine [58]. The block copolymer network was formed to improve the mechanical strength of gels based on carboxymethyl cellulose and the ability to control the release of tetracycline by changing the pH of the medium and temperature. In the oral cavity (pH 2–6) and stomach (pH 1.5–4), tetracycline was not released from the hydrogel, and was only released in the small intestine (pH 7.6). In the temperature range from 25 °C to 33.3 °C, tetracycline was released at a constant rate (18–20%), and when the temperature increased to 39.6 °C, the release rate of tetracycline increased dramatically to 84% (Figure 1).

The developed dual-network hydrogel was non-toxic to L929 mouse fibroblasts, as evidenced by the proliferation of L929 cells on the second day of observation and 85% cell viability on the first day of observation. The low toxicity of the prepared hydrogel system, according to the authors, can be explained by the slow release of tetracycline. The authors found an increase in the swelling rate of the dual-network hydrogel compared to the carboxymethyl cellulose hydrogel in deionized water. The authors explain this fact by the macroporous structure of the hydrogel and a decrease in the electrostatic repulsion between the –COO groups of the side chain of carboxymethyl cellulose. The authors conclude that the hydrogel is promising for use in pharmacology for the “smart” delivery of antibiotics to the small intestine.

Hydrogels are also used to treat inflammatory diseases of the colon (Crohn’s disease and ulcerative colitis) as a drug delivery system. Hydrogels allow reducing the dosage of drugs and, as a result, reducing side effects. For example, the authors [59] demonstrated a prolonged pH-sensitive release of curcumin from hydrogels based on hyaluronic acid, gelatin, and carboxymethylchitosan. The advantage of the work [59] compared to the study [58] is the presence of an in vivo study conducted on mice with DSS-induced colitis (control). The authors found, when the gel was administered orally, a decrease in the concentration of blood serum inflammatory markers IL-6 and TNF-α in the callus of mice in comparison with the control group, which indicated a decrease in the level of inflammation (Table 1).

The use of hydrogels for drug delivery in the treatment of fibrotic skin diseases (scleroderma, hypertrophic scars), infectious skin diseases (leishmaniasis), atopic dermatitis, psoriasis improves their therapeutic effect, reduces systemic toxicity and the cost of treatment. In [60], a differentially crosslinked hydrogel system based on collagen (pepsin) and polyethylene glycol was used to deliver a multidrug system consisting of trichostatin A and transforming growth factor β. A slow release of drugs from the core-shell gel matrix was established for 10 days, which indicates the antifibrotic potential of the gel. However, only about 30% of trichostatin A and about 50% of transforming growth factor β were released in this case.

In [61], a hydrogel made from N,N,N-trimethylchitosan, polyethylene glycolated hyaluronic acid and poloxamer 407 released 86.5% of gallic acid used to treat atopic dermatitis. The authors found that the kinetics of gallic acid release is described by a first-order rate model depending on the initial concentration of gallic acid in the gel, and by the Higuchi rate model, which assumes a diffusion mechanism of drug release.

Transdermal delivery is a simple and non-invasive drug delivery method for the treatment of skin diseases [62]. In works [63,64], 87% of the antifungal drug amphotericin B was transdermal released from a hydrogel based on radiation-crosslinked poly-N-vinylpyrrolidone within 48 h. The drug effectively inhibited the growth of *L. amazonensis promastigotes*. Radiation crosslinking with an irradiation dose of 15 kGy did not affect the stability of the drug. The authors explain the antifungal effect of the drug by its binding to ergosterol, which is a component of the cell membrane of the fungus, and the subsequent leakage of the contents of the fungal cell. The use of hydrogel made it possible to reduce the toxicity of the drug for patients with cardiovascular and kidney diseases.

The studies [59,60,61,63,64] did not consider the penetration of drugs through the skin ex vivo. In [65], a slight absorption of drugs for the treatment of psoriasis (fluocinolone acetonide and salicylic acid) from a gel based on carbopol 934 through the skin of a pig ear into the systemic circulation was determined. In [65], the mechanism of drug release from the gel matrix was studied and it was found that the diffusion-controlled Higuchi model is the most suitable for describing the kinetics of the release of fluocinolone acetonide, and the zero-order model for the kinetics of the release of salicylic acid.

## 3. Gels in Ophthalmology

The use of nano-sized hydrogels for drug delivery using eye drops in ophthalmology can extend the retention time of eye drops and improve their stability. This is because usually eye drops without the proper delivery system are washed away by tear secretions. In addition, eye drops for the treatment of eye diseases without a gel matrix have low bioavailability and several side effects [66].

**Figure 2:**
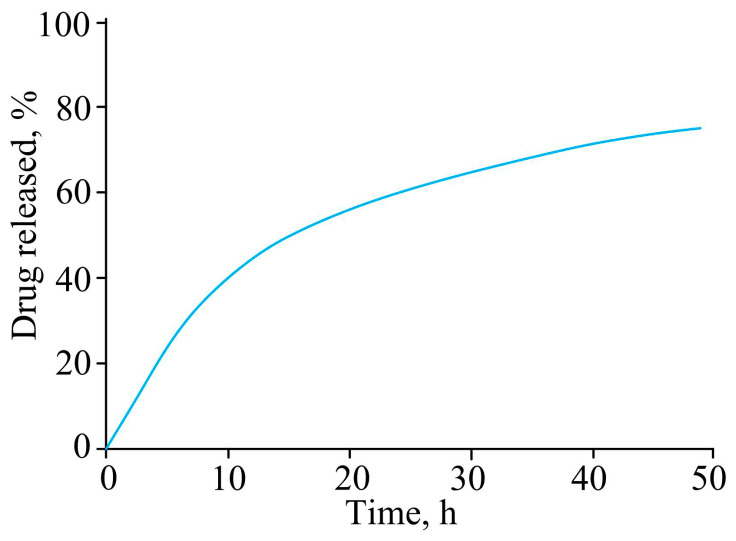
Dexamethasone release from nanogel at 34 °C.

Wen Y. et al. [67] extended the residence time of dexamethasone in the cornea by incorporating it into a nano-hydrogel based on kolliphor P188, kolliphor P407, and polycarbophil. The nano-hydrogel was obtained by the cold solution method at 4 °C. The hydrogel showed sustained release of dexamethasone over 48 h, and released more than 70% of dexamethasone (Figure 2).

**Figure 3:**
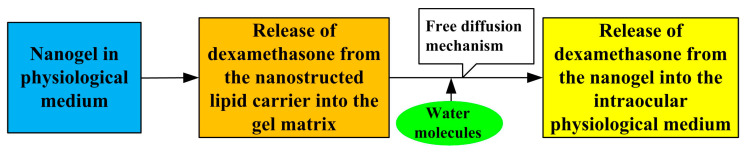
Dexamethasone release mechanism.

The authors explain the prolonged release of the drug by the fact that the drug had to pass through a long labyrinth-like channel in the gel before reaching the cornea of the eye. The release mechanism of dexamethasone consists of two steps, which are detailed in Figure 3. The in vivo study of the gel on the cornea of New Zealand rabbits showed that the nanogel adheres tightly to the surface of the eye due to strong adhesion, which makes it possible to resist the effect of tear fluid on the drug.

Hydrogels based on carboxymethyl agarose and chitosan showed a temperature-sensitive release of the hydrophilic drug diclofenac sodium. In this case, toxic crosslinking agents were not used to prepare the resulting hydrogel, instead of this, the method of coacervation of polymers with oppositely charged functional groups (carboxylate and ammonium groups) was carried out. The formation of hydrogen bonds, electrostatic and dipole-dipole interactions between the ionized amino and carboxyl groups of diclofenac sodium, and the charged centers of the polymer contributed to the macromolecular encapsulation of diclofenac sodium. At pH 6.0 and a temperature of 37 °C, approximately 67% of the drug was released, while at a temperature of 25 °C, only 40% of the drug was released in comparison [68]. According to the authors, this result can be explained by the increase in the mobility of polymer chains at a higher temperature. Using Peppas-Korsmeyer regression, the authors found that the predominant mechanism of drug release is the Fick diffusion mechanism through the gel pores compared to surface release, solvent-controlled release, erosion of polymer degradation. The resulting gels did not have a cytotoxic effect on human keratinocytes (HaCaT cell line), as evidenced by the preservation of cell morphology after 72 h of observation.

However, the disadvantage of this work is the lack of in vivo data on the effect of metabolites formed during the degradation of the nanogel on the eye tissues of rabbits, which is important for confirming the safety of nanogels.

In study [69], along with the prolonged release of diclofenac sodium ophthalmic preparation from a thermosensitive gel based on poloxamer 407 and poloxamer 188 and containing carbon dots 90 nm in size to impart an antibacterial effect, in vivo studies were carried out on the eye tissues of albino rabbits. The authors found that the gel did not cause tissue irritation during 7 days of continuous use. This was evidenced by the absence of conjunctival hyperemia, corneal clouding and inflammation. The authors of [70] also found the absence of conjunctival redness in rabbits for gels based on gellan gum and carbopol 934 containing travoprost, which is intended to reduce intraocular pressure in glaucoma. Based on an in vivo study, the authors of works [69,70] conclude that gels are safe for the treatment of eye diseases.

Traumatic optic neuropathy after an eye injury is one of the diseases in the field of ophthalmology, leading to loss of vision. Alginate gels are an effective means of delivering drugs capable of scavenging reactive oxygen species using a calibration needle. In [71], hydrogels based on calcium alginate containing the antioxidant methylene blue were studied. The hydrogels released more than 50% of the drug over 5 days followed by a slow sustained release of more than 80% of the drug. The hydrogels were non-toxic to human retinal pigment epithelial cells, as evidenced by a cell viability of over 90%. The gels were easily injected in a liquid state with a needle and solidified in situ in the retrobulbar space behind the globe of the eye. The authors conclude that it is possible to use gels for the local controlled release of scavengers of reactive oxygen species without damaging the eye. However, the authors did not conduct in vivo studies of hydrogels in rabbits.

The authors of [72] tested a nanocomposite hydrogel based on poly(hydroxyethyl methacrylate), chitosan, and zinc oxide nanoparticles in vivo on New Zealand white rabbits. The nanocomposite gel contained a drug system that included epigallocatechin gallate, hyaluronic acid nanoparticles, β-1.3-glucan, and SB431542 potent and selective inhibitor of the transforming growth factor-β. The release of epigallocatechin gallate from the gel occurred in accordance with case-II transport due to swelling and relaxation, the release of β-1.3-glucan was regulated by Fick diffusion, in which the diffusion rate is much lower than the relaxation rate, and, finally, the release of SB431542 was regulated by non-Fick anomalous diffusion, in which the diffusion rate is comparable to the rate of relaxation. According to the authors, the gel can be used to restore damaged cornea tissues and prevent scarring, which is important in the pharmacological treatment of multifactorial eye diseases. The degree of tissue recovery after the damage was more than 90%, which is eight times greater than that of eye drops. The gels simultaneously protected the damaged eye from exposure to UV light due to the presence of zinc oxide nanoparticles.

## 4. Gels in Cancer Treatment

Targeted delivery of anticancer drugs with minimal side effects and prolonged release is an important direction in targeted therapy of oncological diseases, including those for local non-invasive drug delivery to the skin for the treatment of melanoma [73], local injection into complex anatomical areas after surgical removal of tumor tissues [74]. The use of gels makes it possible to reduce the side effects of cytotoxic drugs and provide high loading efficiency and therapeutic efficacy. In work [75], an organogel based on lipiodol and 12-hydroxystearic acid organogelator stabilized with polyoxyethylene hydrogenated castor oil (surfactant) was used to deliver the hydrophobic and poorly water-soluble drug paclitaxel to tumor cells. Compared to nanoemulsions, the organogel is more stable and provides sustained release of paclitaxel to B16F10 melanoma cancer cells. The resulting gel showed biocompatibility with primary rat hepatocytes in vitro. The authors explain the biocompatibility of the organogel by the content of unsaturated fatty acids in it, which contribute to maintaining the function of hepatocytes without changing their morphology.

It is known that the agglomeration of nano-sized drug formulations leads to a decrease in their therapeutic effect. The hydrogel matrix prevents the agglomeration of polymer nanoparticles containing anticancer drugs. In work [76], to reduce the harmful effects on healthy cells, doxorubicin and dasatinib were introduced into a gelatinous matrix with porosity and biocompatibility. However, the disadvantage of this study [76] is the lack of data on the viability of cancer cells.

In studies [77,78,79], on the contrary, data on the viability of cancer cells are presented. The authors of [77] studied the effect of spherical nanogels based on poly-N-isopropylacrylamide containing the anticarcinogenic drug 5-fluorouracil and the photosensitizer indocyanine green on the viability of HeLa cells. For intracellular targeted drug release, laser radiation was used, which destroyed the nanogel and facilitated the release of the drug and photosensitizer into the cell cytoplasm. The viability of HeLa cells decreased to 40% (Table 2).

The authors explain this fact by the release of a large amount of heat and the generation of singlet oxygen in the cancer cell. The developed thermosensitive nanogel, according to the authors, can be used in combined chemo- and photodynamic therapy, and will also reduce the side effects of chemotherapy.

The authors of [78] studied the effect of a radiation-crosslinked pH-sensitive nanogel based on polyethylene glycol and polymethacrylic acid containing previously unstudied N-(4-(N-(diaminomethylene)sulfamoyl)phenyl)carbamothioyl)acrylamide on the viability of four cancer cell lines (HepG2, A549, MCF-7, HCT-116). The authors found that the synthesized drug has better antitumor activity compared to the commercial antitumor drugs sorafenib and erlotinib. Nanogel showed no hepatotoxic effect, unlike the indicated anticancer drugs. Unlike erlotinib, the drug showed a slow rate of total clearance, which, according to the authors, indicated a longer duration of its action.

The authors of [79] showed that a nanogel based on di(ethylene glycol) methyl ether methacrylate, poly(ethylene glycol) methyl ether methacrylate, and hyaluronic acid methacrylate copolymers crosslinked with di(ethylene glycol) diacrylate effectively releases low doses of antitumor drug preparations of doxorubicin and mitoxantrone under the action of the enzyme hyaluronidase on the nanogel. Drugs were loaded into the gel using electrostatic interactions between the gel carboxyl anions and the drug cation. The drug release profiles in the presence of the enzyme were sigmoidal, with 60% of the total drug being released by diffusion. The authors found that the kinetics of drug release with the participation of the enzyme is described by a zero-order model. According to the authors, this is due to the acceleration of diffusion during enzymatic degradation. The authors established the cytotoxic activity of the nanogel against human breast cancer cells MCF-7 and ovarian cancer cells A278, and the absence of cytotoxic activity against healthy MCF-10A and HOF cells. This was evidenced by low IC_50_ values, close to zero.

For the treatment of malignant neoplasms of the peritoneum, intraperitoneal delivery of chemotherapeutic drugs using hydrogels is more effective than intravenous treatment. In [80], the ability to dilute in the abdominal cavity of a gel based on covalently crosslinked polyethylene glycol containing nanocapsules was studied. The nanocapsules were loaded with docetaxel. As a result, the authors found that the gels are resistant to dilution in a buffer that simulates the intra-abdominal environment and retained their integrity for 2–3 weeks. The authors explain this fact by covalent rather than physical cross-linking of polyethylene glycol, which contributes only to swelling, without dissolution. In vivo testing of the gel in mice using a fluorescent marker showed retention of the gel in the abdominal cavity for 24 h. According to the authors, the gel can be used by surgical oncologists for implantation into the abdominal cavity at the site of tumor resection.

Compared to chemo- and photodynamic therapy, magnetic thermo-sensitive therapy of tumors under the action of a high-frequency alternating magnetic field using polymer hydrogels containing magnetic nanoparticles and antitumor drugs has fewer side effects and is more effective [81]. It is known that normal and tumor cells have different sensitivities to temperatures. Tumor cells, unlike normal ones, die at temperatures in the range of 42–46 °C.

The use of the double emulsion method produced core-shell gels makes it possible to encapsulate both hydrophilic and hydrophobic anticancer drugs in them. Researchers from China [81] loaded hydrophobic camptothecin and hydrophilic doxorubicin hydrochloride into a gel based on a copolymer of N-isopropylacrylamide and acrylamide (the cross-linking agent is methylene-bis-acrylamide) and 0.4 wt.% magnetite nanoparticles. Under the action of a magnetic field, the gels were heated and underwent a phase transition. As a result, the absorbed water in the shell and the hydrophilic drug were released. The hydrophobic drug, located in the core, was released due to the formation of ruptures in the hydrogel shell under the influence of a magnetic field. The authors investigated the therapeutic effect of drugs and found that the viability of liver cancer cells (cell line HepG2) in the combined treatment with camptothecin and doxorubicin hydrochloride is only 7.8% which is evidence of this method’s efficiency.

In work [82], hydrophilic doxorubicin hydrochloride was loaded into a magnetic thermo-sensitive hydrogel based on polyvinyl alcohol, poly-N-isopropylacrylamide, and polyacrylic acid. The drug encapsulation efficiency was 87% due to the strong physical interaction between the carboxyl anions of polyacrylic acid and the ammonium cations of doxorubicin. In contrast to the investigation [81], in [82], the therapeutic effect of the drug was evaluated in monochemotherapy and in combination with chemotherapy with magnetic field exposure (hyperthermia) in relation to MCF-7 breast cancer cells after 48 h of observation. The authors showed that combination therapy leads to higher antitumor activity compared to chemotherapy. However, the decrease in the viability of cancer cells is small—no more than 15%.

The disadvantage of magnetic therapy is the difficulty of uniform dispersion of magnetic nanoparticles in the gel matrix, without their aggregation, which leads to their uneven heating. The temperature inside the gel must be accurately controlled to ensure stable drug release and maintain drug bioactivity.

The study of the mechanism of interaction of anticancer drugs with a hydrogel matrix is of interest for the development of new drug delivery systems. The authors of [83] found that the anticancer drugs gemcitabine and doxorubicin predominantly react with a gel matrix based on glycolchitosan and bifunctional polyethylene glycol using the van der Waals adsorption mechanism and with the formation of hydrogen bonds, respectively. To simulate interactions, the authors used the atomistic model method and the Materials Studio Amorphous Cell software package. Van der Waals interactions of gemcitabine and gel accounted for 58.4%, while the rest was for π-stacking interactions and interactions involving hydrogen bonds.

## 5. Gels in Dentistry

**Figure 4:**
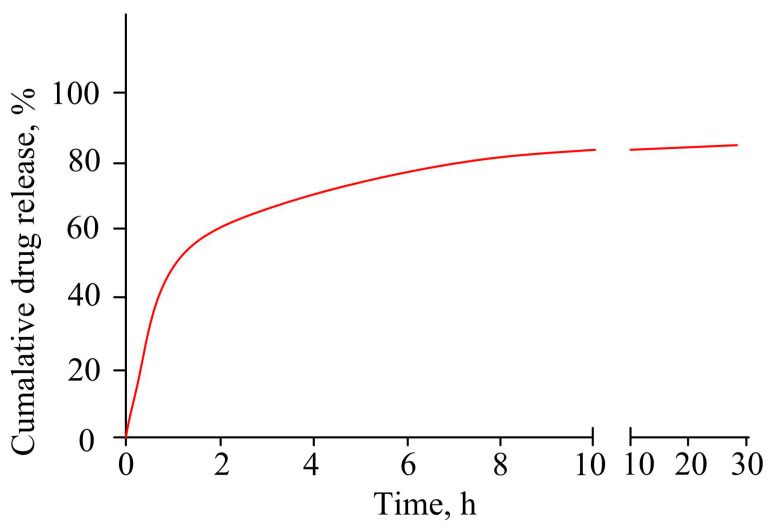
In vitro release of metronidazole from gel into phosphate buffer.

Localized release of drugs from polymer hydrogels for the treatment of periodontitis avoids side effects caused by high doses of drugs—gastrointestinal intolerance, toxicity. Hydrogel systems make it possible to deliver buccal preparations to the periodontal pocket mucosa by prolonged contact with the buccal mucosa. The authors of [84] studied the therapeutic potential of a hydrogel based on hydroxyethyl cellulose containing the antibiotic metronidazole embedded in solid lipid nanoparticles. For the study, the mucous membrane of the buccal bone of a pig in phosphate buffer (pH 6.8) was taken. It has been established that from 173 to 321 µg/cm^2^ of the drug penetrates through the membrane within an hour. The gel had antibacterial activity against *S. aureus* and *E. coli*. The developed gel showed a prolonged release of metronidazole during the day (Figure 4). The authors explain the epy burst release of the drug by adsorption of the drug on the surface of lipid nanoparticles and defective encapsulation of the drug inside the particles.

The delayed release of the drug, according to the authors conclusions, was provided by the hydrogel structure of hydroxyethylcellulose. The authors come to the conclusion that the gel is a promising tool for delivering antibiotics to the periodontal pocket.

In the following study [85], methylcellulose-based gels containing atorvastatin and rosuvastatin were tested in vivo on patients with periodontitis. Statins had osteoblastic properties and prevented alveolar bone resorption, as evidenced by a significant improvement after 6 months of gel treatment. The authors conclude that gels can be used in clinical practice in the field of non-surgical periodontal therapy for local delivery of statins in the periodontal pocket.

However, the works [84,85] did not study the processes of alveolar bone restoration and collagen regeneration. In [86], baicalin and clove oil were loaded into a nanogel based on poloxamers 407 and 188. The gel improved the restoration of periodontal tissues through collagen regeneration and osteogenesis.

## 6. Gels in Tissue Engineering

Hydrogel scaffolds, showing the sustained release of growth factors, are promising materials for the physiological restoration of bone tissue in the field of tissue engineering. The authors of the following study [87] developed a porous composite gel scaffold based on short-chain chitosan and polyethylene glycol. Polyethylene glycol was included in the gel to improve the mechanical properties of chitosan-based gels, since they are brittle and decompose in vivo. The gel demonstrated a sustained release of acetylsalicylic acid for 14 days, which promoted osteogenic differentiation and bone tissue regeneration. An in vivo study of the scaffold in a mouse calvarial bone defect model demonstrated a high level of osteogenesis. However, in [87] there are no data on cell adhesion and viability of osteoblasts.

On the other hand, in work [88], membranes based on polycaprolactone and gelatin obtained by electrospinning were studied in vitro for toxicity to osteoblasts (cell line MC3T3-E1). The authors showed that the membrane does not affect the viability of cells when they are incubated on the membrane for 3 days. The authors suggest using the prepared membranes for the delivery of hydrophilic and hydrophobic active agents for bone tissue regeneration with their slow prolonged release for more than two weeks.

## 7. Gels for the Treatment of Joint Diseases

Treatment of arthritic diseases with the help of gels which contain biological preparations and antibodies can reduce inflammation in the joints and musculoskeletal pain. The inclusion of drugs in the gel matrix reduces the clearance of drugs into the blood capillaries and lymphatic system. Gels based on hyaluronic acid (2400–3600 kDa) and polyvinylpyrrolidone (1000–1500 kDa) provide prolonged release of adalimumab into the joint cavity in osteoarthritis [89]. Adalimumab was attached to the gel using electrostatic interactions with hyaluronic acid. According to the authors, this fact limits the diffusion of adalimumab from the gel. A gel based on chitosan, N-isopropylacrylamide copolymer and 1,4-butanediyl-3,3′-bis-1-vinylimidazolium BBVIm ionic liquid and carbon nanotubes was able to respond to electrical and thermal stimulation. But the developed gel released only 37% of the hydrophobic drug ketoprofen, intended for the treatment of musculoskeletal pain [88]. Moreover, the authors of [89,90] did not investigate the toxicity of gels.

A hydrogel based on oligosaccharide, chitosan alginate, norbornene, and tetrazine released approximately 84% of ketoprofen into the fluid simulating the gastrointestinal tract after 24 h and was nontoxic to normal HEK-293 cells [91]. Cell viability was approximately 95%. At pH 7.4, 96% of the drug was released, while at pH 2.2, no more than 45% of the released drug was detected. The pH-sensitive release is attributed to the formation of hydrogen bonds between carboxyl and hydroxyl groups at acidic pH and the repulsion of carboxylate anions at pH 7.4, stretching the alginate chains and enhancing the penetration of liquid into the gel. The hydrogel was highly biodegradable due to the enzymatic cleavage of β-1.4-glycosidic bonds of polysaccharide chains. However, it is necessary to study further the effect of gels on the growth of bone tissue cells.

**Figure 5:**
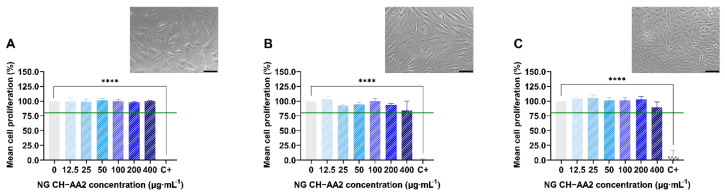
Relative proliferation and morphology of chondrocytes (**A**), synoviocytes (**B**), and osteoblasts (**C**) treated with nanogel NG CH-AA2 for 72 h. CH—chitosan, AA2—acetic acid, 2% *v*/*v*. **** *p* < 0.0001 (one-way ANOVA analyses). Scale bars: 100 µm [92].

The effect of chitosan-based nanogels on chondrocytes, synoviocytes, and osteoblasts in patients with osteoarthritis was studied in [92]. The authors found that the proliferation of these cells exceeds 80% at any concentration of nanogels (Figure 5), and the integrity of the cell membrane is maintained when exposed to nanogels for 72 h.

Therefore, the developed nanogels are safe in relation to osteochondral cells. However, the obtained in this work gels showed moderate genotoxicity.

Hyaluronic acid-based gels containing exosomes of endothelial cells are used to repair bone fractures. This is explained by the anti-inflammatory and antibacterial action of hyaluronic acid and the ability of exosomes to differentiate anti-osteoclasts [93]. Hydrogels based on hydroxypropylchitin and porous chitosan microspheres are effective for repairing articular cartilage defects. In [94], it was shown that these hydrogels containing the drug dimethyloxalylglycine regenerate hyaline cartilage and subchondral bone. In the work, in vivo studies were carried out, consisting of subcutaneous implantation of the material in rats in the area of the bone and cartilage defect. The authors found faster defect closure after 6 weeks of follow-up compared to the control group (saline). However, the authors do not explain why they used gels and porous microspheres for cartilage regeneration rather than porous hydrogel scaffolds.

For the treatment of infectious bone diseases, in particular osteomyelitis, gel delivery vehicles for antibiotics or osteoinductive factors based on biopolymers, such as silk fibroin, sodium, or calcium alginate, are shown to be effective. These delivery systems are biodegradable, non-toxic, and do not cause antibiotic resistance. A gel based on silk fibroin and sodium alginate containing the antibiotic teicoplanin and phenamyl to induce osteogenesis showed a pH-sensitive behavior [95]. At three pH values of 5.5, 7.4, and 8.5, the percent release of the antibiotic was 83, 90, and 89%, respectively, within 35 days. The authors explain the pH-dependent release of the drug by the conversion of alginate carboxyl groups into COO^−^ anions at alkaline pH 7.4 and 8.5 and by an increase in the degree of swelling of the gels due to the electrostatic repulsion of polymer chains. The hydrogel was biocompatible with rabbit osteoblast cells, since it did not adversely affect their viability. The gel showed the ability for osteoblast adhesion and proliferation, as well as antibacterial activity against *methicillin-resistant S. aureus*, which causes osteomyelitis. Therefore, the resulting gel is a promising material for the treatment of bone infection.

## 8. Gels for the Treatment of Neurodegenerative Diseases

The polysaccharides κ-carrageenan and chitosan, containing anion-sulfate and amino groups, respectively, provide electrostatic interactions with drugs used to treat neurodegenerative diseases—Alzheimer’s disease and Parkinson’s disease. In [96], rivastigmine was incorporated into a nanogel based on chitosan and κ-carrageenan. The κ-carrageenan was added to chitosan to reduce the solubility of chitosan in the acidic environment of the stomach. Nanogel released more than 90% of the drug into a phosphate buffer (pH 7.4), simulating the intestinal environment. Drug release was controlled by Fickian diffusion. The polymer matrix protected the drug from degradation in the stomach (pH 1.2). The developed nanogel was non-toxic for human fibroblast cells, as was shown by the cell viability of more than 88%.

To deliver rivastigmine to the intestinal environment (pH 7.4), gels based on polyvinyl alcohol, gum arabic aldehyde as a non-toxic cross-linking agent, and graphene oxide are used. Graphene oxide is added to improve the mechanical strength and degree of swelling of gels due to the formation of hydrogen bonds between carboxyl and hydroxyl groups on the surface of sheets of graphene oxide nanosheets with hydroxyl groups of polyvinyl alcohol. In [97], the degree of swelling of gels containing 0.11 wt.% graphene oxide increased by about 600% compared to gels without graphene oxide nanosheets, and the tensile strength increased by about 58%. However, when a larger amount of graphene oxide was added, the degree of swelling decreased, which the authors attribute to nanosheet aggregation.

It is known that nasal delivery of drugs makes it possible to achieve faster absorption compared to oral delivery and bypasses the blood-brain barrier when delivering drugs to the brain [98,99]. Instead of nanogels, which are administered orally, for invasive drug delivery through the nasal cavity, thermosensitive gels are used, which provide a slow release of drugs into the nasal cavity. In study [100], rhynchophylline was delivered to the brain through the blood-brain barrier using a delivery system based on poloxamer 407, poloxamer 188, polyethylene glycol-6000, and hydroxypropyl-β-cyclodextrin. After intranasal administration, the solution of these substances turned into a gel-like state at a physiological temperature range of the nasal cavity of 32–34 °C. Hydroxypropyl-β-cyclodextrin was added to improve the absorption of the gel in the nasal cavity. The developed thermosensitive gel did not have side effects on the body and nasal mucosa of mice. However, the disadvantage of the resulting gel is its cytotoxicity against SH-SY5Y and bEnd.3 cells at high concentrations (160 μg/mL or more). An in vitro study of the toxicity of a gel based on L-glutamine amide and benzaldehyde for intranasal delivery of L-DOPA preparation into the nasal cavity with respect to human nasal epithelial cells also showed a decrease in cell viability [101]. According to the authors, the toxicity was due to the amphiphilic glutamine amide units in the gel.

## 9. Gels for the Delivery of Antimicrobial and Antipyretic Drugs

Gels based on biocompatible synthetic polymers are effective delivery vehicles for antimicrobial and anti-inflammatory drugs. pH and temperature-sensitive gels of copolymers of acrylic acid and N-isopropylacrylamide, having a porous structure and a degree of swelling in the water of approximately 11,000%, is a promising means of delivering ibuprofen. This was evidenced by the data of modeling the adsorption-diffusion of ibuprofen from the gel matrix [102]. However, the percentage release of ibuprofen did not exceed 50% at pH 7.4 and did not exceed 70% at pH 2.0. In contrast to [102], in [103], the percentage of ibuprofen release from a porous hydrogel based on a copolymer of N-isopropylacrylamide and crotonic acid was more than 50% at pH 6.8, but not more than 11% in a 0.1 M HCl solution.

The authors of investigation [104] found that more than 80% of the antibiotics cefuroxime, tetracycline, amoxicillin, and the anti-inflammatory agent acetylsalicylic acid are released from gels based on hyaluronic acid under physiological conditions (phosphate buffer solution, 37 °C). The gels had an antibacterial effect against *S. aureus*, which causes clinical infections, and were non-toxic in experiments in vitro and in vivo. Gels loaded with amoxicillin reduced the viability of *S. aureus* pathogens by 99.9% after 6 h of observation. However, after 24 h the viability decreased by only 57%. The authors explain this fact by the production of the enzyme beta-lactam penicillinase by bacteria, which increases the resistance of bacteria to the antibiotic. The resulting injectable hydrogels, according to the authors, can be used to treat bacterial infections and inflammatory reactions.

Gel matrix antivirals can be used to treat COVID-19. The authors of [105] established the ability of a porous gel based on natural laponite clay and physically cross-linked polyvinyl alcohol loaded with rifampicin to inhibit the 3CL^pro^ protease of the SARS-CoV2 virus and block the attachment of the S-protein to the ACE2 cell target receptor. The authors explain the therapeutic effect of rifampicin by the ability to form hydrogen bonds with amino acid residues of the S-protein. However, high concentrations of polyvinyl alcohol led to the formation of a dense structure, due to physical interactions with hydroxyl groups and amino groups of rifampicin, and prevented the release of the drug.

## 10. Synthetic and Natural Polymers for Gels Drug Delivery Systems

Natural polymers are promising matrices for the manufacture of gel drug delivery systems because they are biocompatible and biodegradable in the human body. In particular, alginate hydrogels decompose by ion exchange [71]. Silk fibroin and collagen are degraded to form amino acids or peptides with a low molecular weight that are nontoxic to the body. Hyaluronic acid is degraded in the human body by the action of the enzyme hyaluronidase, which breaks down hyaluronic acid into monosaccharaides by breaking glycosidic β-1,4 bonds [79,106]. Due to their ability to biodegrade, natural polymers are non-toxic, do not accumulate in tissues, and are excreted from the body as degradation products [107].

Synthetic carbon chain polymers, in particular polyacrylic and polymethacrylic acids, polyvinyl alcohol, are non-biodegradable, since they contain hydrolytically stable C-C bonds in the main chain. Carbon chain polymers are excreted from the body, through the kidneys, and high molecular weight fractions clog the renal tubules. For example, high molecular weight poly-N-vinylpyrrolidone can accumulate in tissues due to a lack of glomerular filtration and has low biodegradability [89]. Low molecular weight poly-N-vinylpyrrolidone (less than 25 kDa) is excreted via the kidneys. Studies in rats, pigs, dogs, and rabbits have shown the non-toxicity of orally administered poly-N-vinylpyrrolidone. Diarrhea may be the only side effect when administered in high doses [107].

Biodegradable synthetic polymers include heterochain polymers containing urethane, urea, ester, and amide groups. The destruction of the polymer chain proceeds under the action of proteolytic enzyme systems. Heterochain polyethylene glycol is capable of being oxidized with the formation of toxic products; it is antigenic and immunogenic [108].

## 11. Crosslinkers for Gels Preparation

Unlike physically cross-linked gels, chemically cross-linked gels can be toxic to the human body due to the entry into the bloodstream of toxic, carcinogenic and teratogenic residual cross-linking agents, such as glutaraldehyde, and the occurrence of immunological reactions on them. Therefore, to use chemically cross-linked gels in pharmacology, it is necessary to purify ready-made gels from low-molecular-weight cross-linking agents using dialysis or use pre-synthesized non-toxic and biodegradable polysaccharide cross-linking agents [97], biodegradable cystamine containing disulfide bonds [109], and genipin for cross-linking of collagen, gelatin, chitosan and its derivatives [110], tetraethyl orthosilicate and graphene oxide for crosslinking of sodium alginate [111], tetraethyl orthosilicate for crosslinking of chitosan and guar gum with hydroxyl groups [112], vanillin and citric acid for crosslinking chitosan [113] and others. The use of more reactive cross-linking agents makes it possible to achieve a greater degree of cross-linking of the polymers, which leads to a lower content of the residual amount of the cross-linking agent in solution.

## 12. Conclusions

The considered in current review results testify to the increased possibilities of modern chemistry, biochemistry and nanotechnology in terms of creating gel systems capable of releasing a biologically active substance according to a predetermined program, in the right place, at the right concentration, and at the right time, that is, simulating individual functions of living tissues and organs.

Modified polymeric hydrogels, including nanogels, which not only play the role of a bioinert matrix, but also perform regulatory, catalytic, and transport functions due to the active chemical fragments introduced into them, can significantly advance the solution to the problem of directed drug transport. This will significantly reduce the toxicity of drugs and expand the range of their medical application.

Much greater possibilities for controlling the rate of release of active pharmaceutical agents are opened when using hydrogels and nanogels that can change the degree of swelling with changes in environmental parameters, such as temperature, pH, or chemical composition.

It is expedient to use such systems for highly active, rapidly metabolizing drugs intended for the treatment of chronic patients for a long time, as well as for the correction of undesirable acute conditions. Therefore, the most promising will be the creation of polymer gel systems, containing immobilized antitumor and cardiovascular drugs, drugs acting on the central nervous system, hormones, prostaglandins, vitamins, etc.

According to the analyzed articles, the largest number of articles published in 2021–2022 is devoted to the study of drug release from different types of gels (52 articles). In second place in terms of prevalence are parameters such as in vitro toxicity to normal human or mouse cells and the therapeutic effect of the released drug (25 articles). A smaller number of articles is devoted to the consideration of the degree of swelling of gels and in vivo gels studies (16 articles). Therefore, for the application of polymer gels in clinical practice as drug delivery systems, additional in vivo studies are required, which are a criterion for the safety of such systems and preparations for humans.

In the reviewed articles, much attention is paid to the study of the kinetic patterns of drug release from gels, the establishment of a release profile, and models that describe kinetic curves. However, the effect of the polymer matrix on the drug release profile and the determination of the nature of the physical interactions of drugs with the polymer matrix using spectral methods require further study. In addition, in the reviewed articles, there is not much data on mechanisms for the influence of drug-loaded polymer gels on normal human cells. The study of such mechanisms could help to reduce their cytotoxic effect in final preparations.

## Figures and Tables

**Table 1:** Mouse blood serum concentration of anti-inflammatory cytokines.

Cytokine	Concentration (Control), pg mL^−1^	Concentration (Curcumin/Gel), pg mL^−1^
IL-6	17.0070 ± 0.5005	10.3452 ± 1.6804
TNF-α	31.0771 ± 6.5353	11.9864 ± 5.3004

**Table 2:** Viability of HeLa cells depending on the concentration of nanogel with drug and photosensitizer (Dulbecco’s modified Eagle’s medium, containing 10% fetal bovine serum and 1% penicillin-streptomycin).

Concentration, µg mL^−1^	Relative Cell Viability, %
0 (control)	100
10	50 ± 5
30	47 ± 5
50	43 ± 5
80	41 ± 5
100	40 ± 5

## Data Availability

Not applicable.
